# The CanPath-HDRN Canada Collaboration: Enabling Multi-jurisdictional Research in Canada

**DOI:** 10.23889/ijpds.v9i5.2813

**Published:** 2024-09-18

**Authors:** Jennifer Brooks, Anne Hayes, Riaz Alvi, Mahmoud Azimaee, Parveen Bhatti, Sheraz Cheema, Tim Choi, Nouar ElKhair, Megan Fleming, Noah Frank, Katelyn Frizzell, Jodi Gatley, Lindsey Gilbert, Simon Gravel, Shandra Harman, Jason Hicks, Vikki Ho, Jordan Hunt, Stefana Jovanovska, Victoria Kirsh, Robyn Kydd, Carmen La, Kendra Lester, Guillaume Lettre, Magda Nunes De Melo, Mary-Ann Standing, Lindsay Stewart, Donna Turner, Robin Urquhart, Jennifer Vena, Eric Youngson, Philip Awadalla

**Affiliations:** 1 CanPath; 2 University of Toronto; 3 Health Data Research Network Canada; 4 Healthy Future Sask; 5 ICES; 6 BC Generations Project; 7 Population Data BC; 8 Health Data Nova Scotia; 9 New Brunswick Institute for Research, Data and Training (NB-IRDT); 10 CARTaGENE; 11 Alberta’s Tomorrow Project; 12 Atlantic PATH; 13 CIHI; 14 Ontario Health Study; 15 Secure Island Data Repository (SIDR); 16 NL Health Serv5; 17 Manitoba Tomorrow Project; 18 Alberta Health Serv5

## Objective

To highlight the partnership between the Canadian Partnership for Tomorrow’s Health (CanPath) and Health Data Research Network (HDRN Canada), which enables researchers to link CanPath’s health and lifestyle survey data to health records and related data across multiple regions.

## Background

CanPath is a population health study of over 350,000 Canadians from seven regional cohorts across all ten provinces, making it one of the world's largest population cohorts. HDRN Canada is a network of member organizations, including provincial, territorial, and pan-Canadian data centres.

## Approach

This partnership leveraged collective expertise and resources to facilitate the linkage of CanPath’s harmonized data to multi-regional health and health-related administrative data held at HDRN Canada’ s data centres. Researchers can access these data for multi-regional projects through HDRN Canada’s Data Access Support Hub, which provides a single access portal.

## Results

Researchers were able to successfully link CanPath’s survey data to provincial administrative health data. The collaboration’s streamlined process for data access enhances efficiency and facilitates pan-Canadian population health research.

## Conclusion

This collaboration demonstrates the feasibility and value of linking population health datasets while demonstrating the challenges and opportunities associated with accessing national administrative health data within a federated health data system.

## Implications

The partnership between CanPath and HDRN Canada has significant implications for advancing population health research in Canada. By providing researchers with access to linked data from diverse sources, the partnership enables comprehensive investigations into health determinants, disease patterns, and clinical outcomes. This enhances the scope and depth of population health research in Canada, thereby leading to a better understanding of the most pressing health challenges.

